# Targeting CXCR4 with [^68^Ga]Pentixafor: a suitable theranostic approach in pleural mesothelioma?

**DOI:** 10.18632/oncotarget.18235

**Published:** 2017-05-27

**Authors:** Constantin Lapa, Stefan Kircher, Andreas Schirbel, Andreas Rosenwald, Saskia Kropf, Theo Pelzer, Thorsten Walles, Andreas K. Buck, Wolfgang A. Weber, Hans-Juergen Wester, Ken Herrmann, Katharina Lückerath

**Affiliations:** ^1^ Department of Nuclear Medicine, University Hospital Würzburg, Würzburg, Germany; ^2^ Institute of Pathology, University of Würzburg, Comprehensive Cancer Center Mainfranken (CCCMF), Würzburg, Germany; ^3^ Scintomics GmbH, Fürstenfeldbruck, Germany; ^4^ Department of Internal Medicine I, Division of Pulmonology, Universitätsklinikum Würzburg, Würzburg, Germany; ^5^ Department of Thoracic Surgery, University Hospital Magdeburg, Magdeburg, Germany; ^6^ Molecular Imaging and Therapy Service, Memorial Sloan-Kettering Cancer Center and Weill-Cornell Medical College, New York, New York, USA; ^7^ Pharmaceutical Radiochemistry, Technische Universität München, Munich, Germany; ^8^ Department of Nuclear Medicine, University Hospital Essen, Essen, Germany

**Keywords:** PET, CXCR4, [^68^Ga] pentixafor, pleural mesothelioma, theranostics

## Abstract

C-X-C motif chemokine receptor 4 (CXCR4) is a key factor for tumor growth and metastasis in several types of human cancer. This study investigated the feasibility of CXCR4-directed imaging with positron emission tomography/computed tomography (PET/CT) using [^68^Ga]Pentixafor in malignant pleural mesothelioma.

Six patients with pleural mesothelioma underwent [^68^Ga]Pentixafor-PET/CT. 2′-[^18^F]fluoro-2′-deoxy-D-glucose ([^18^F]FDG)-PET/CT (4/6 patients) and immunohistochemistry obtained from biopsy or surgery (all) served as standards of reference. Additionally, 9 surgical mesothelioma samples were available for histological work-up.

Whereas [^18^F]FDG-PET depicted active lesions in all patients, [^68^Ga]Pentixafor-PET/CT recorded physiologic tracer distribution and none of the 6 patients presented [^68^Ga]Pentixafor-positive lesions. This finding paralleled results of immunohistochemistry which also could not identify relevant CXCR4 surface expression in the samples analyzed.

In contrast to past reports, our data suggest widely absence of CXCR4 expression in pleural mesothelioma. Hence, robust cell surface expression should be confirmed prior to targeting this chemokine receptor for diagnosis and/or therapy.

## INTRODUCTION

Malignant pleural mesothelioma (PM) is a rare (incidence, 7/1.000.000/year) aggressive cancer associated with occupational exposure to asbestos that is often diagnosed at late, unresectable stages [1, 2]. Despite aggressive treatment algorithms including radiation and/or chemotherapy, the prognosis of mesothelioma has remained poor for decades, with an average survival of 9–12 months [3]. Therefore, new molecular targets for this fatal disease need to be identified.

C-X-C motif chemokine receptor 4 (CXCR4) and its ligand CXCL12 play an important role in a variety of physiological processes that rely on the recruitment and homing of stem cells, progenitor cells and immune cells. CXCR4 is over-expressed in more than 20 human tumor types, promoting tumor growth and progression, tumor invasiveness and metastasis [4]. In malignant mesothelioma, robust overexpression of CXCR4 was reported in human mesothelioma cell lines and the majority of mesothelioma tissues, respectively [5].

Recently, Wester and co-workers developed [^68^Ga]Pentixafor ([^68^Ga]CPCR4.2), a cyclic pentapeptide that enables sensitive and high-contrast imaging of human CXCR4 receptor expression *in vivo* [6–8] . Proof-of-concept visualization with this tracer could be demonstrated for several different hematologic and other neoplasms including leukemia, lymphoma, multiple myeloma, glioblastoma or small cell lung cancer, but also in other (inflammatory) disease conditions, such as stroke and myocardial infarction [9–15]. Interestingly, in a pilot study, the *in vitro* CXCR4 expression profile of solid cancers was shown to be different from the *in vivo* distribution as revealed by CXCR4-targeted PET imaging [16].

The aim of this pilot study was to assess the feasibility of non-invasive imaging of CXCR4 in patients with pleural mesothelioma.

## RESULTS

### Patients

Histopathologic diagnosis had been derived by surgical or biopsy samples in all patients. 4/6 patients suffered from epitheloid, the remaining subjects from desmoplastic/sarcomatoid and microcystic mesothelioma, respectively. All subjects presented with diseases confined to the mesothelial surfaces of the pleural cavity. No extra-pleural metastatic sites were present at the time point of imaging (Supplementary Table 1).

### Surgical samples

The surgical samples available for IHC were derived from patients with epitheloid (*n =* 4), sarcomatoid (*n =* 3), and biphasic (*n =* 2) mesothelioma, respectively. All samples were derived from patients (8 males, 1 female) with the primary diagnosis of PM. None of them presented with extra-pleural metastases.

### Image analysis

**Figure 1:**
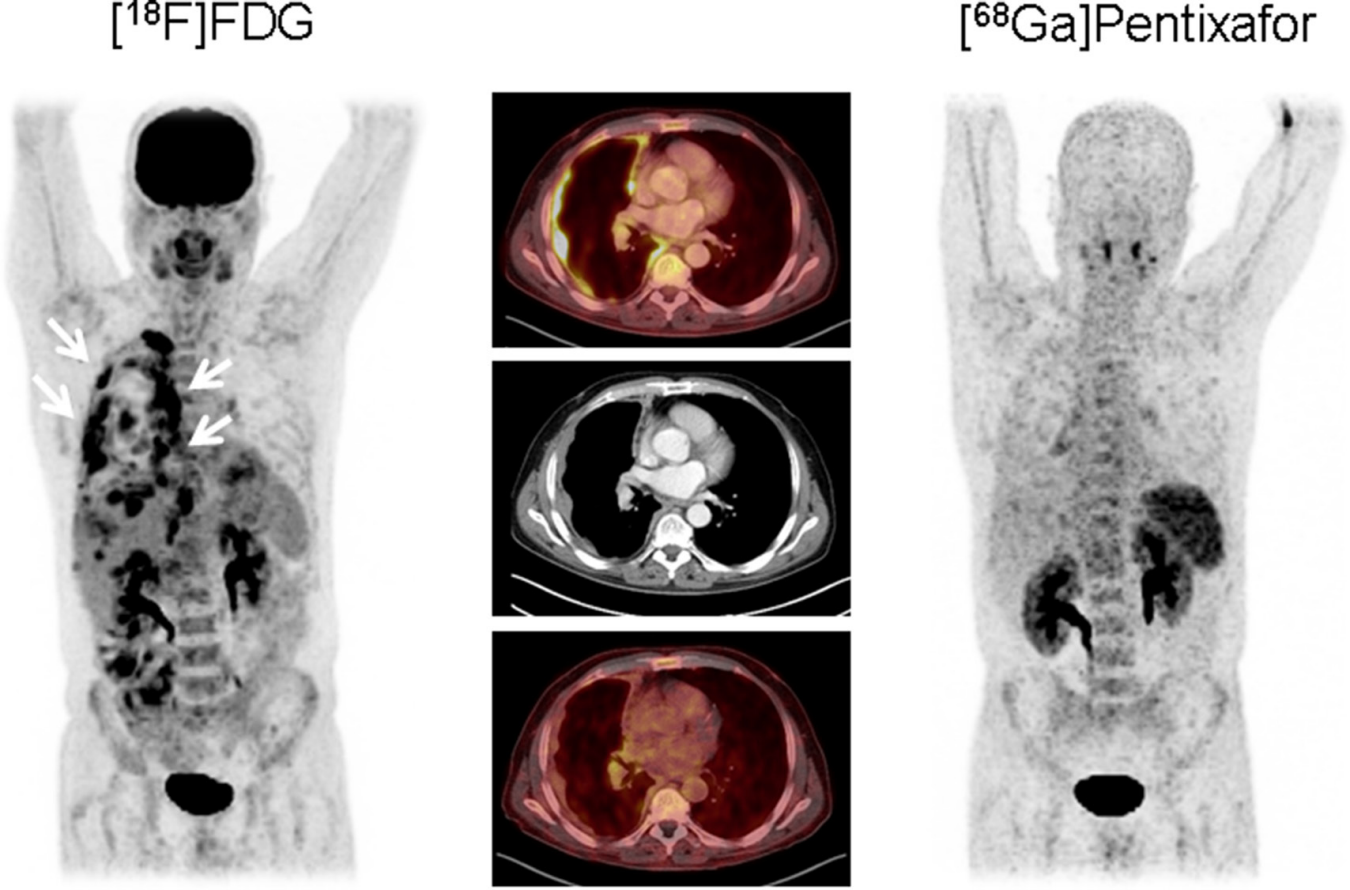
Example of epitheloid mesothelioma (patient #3) without relevant CXCR4 expression Maximum intensity projections (outer columns) and transaxial slices (middle column) of [^18^F]FDG PET/CT (left, upper row of the middle column) and [^68^Ga]Pentixafor PET/CT (right, lower row of middle column) in a patient with the primary diagnosis of epitheloid mesothelioma. The multiple vital tumor lesions along the right pleura detected by [^18^F]FDG PET/CT (arrows) do not express CXCR4. This finding was confirmed by immunohistochemistry.

On visual image analysis of the scans, none of the six patients presented relevant focal [^68^Ga]Pentixafor-positive lesions (Figure 1). Only physiologic tracer distribution was recorded.

In semi-quantitative analysis, SUV_mean_ ranged from 1.4 to 2.9 with a median of 2.5 and SUV_max_ from 2.3 to 4.4 with a median of 3.9, respectively. Median blood pool activity was 1.8 (range, 1.6–2.7) for SUV_mean_ and 3.6 (range, 2.8–4.6) for SUV_max_. Accordingly, tumor-to-blood pool (TBR) ratios were low in all cases with a median of 1.2 (range, 0.9–1.6) for TBR_mean_ and 1.0 for TBR_max_ (range, 0.7–1.4), respectively.

In contrast, [^18^F]FDG-PET identified viable tumor lesions in all patients with SUV_mean_ ranging from 5.8 to 11.3 (median, 8.3) and SUV_max_ from 7.8 to 18.4 (median, 11.5), respectively. With median blood pool uptake of 1.8 (SUV_mean_, range, 1.7 to 2.2) and 2.7 (SUV_max_, range, 2.0 to 3.2), median TBRs were 4.9 (range, 2.9–5.1) for SUV_mean_ and 5.2 (range, 2.5–5.8) for SUV_max_, respectively. Results for each individual patient are mentioned in Table 1.

**Table 1:** Individual imaging results

No.	Age	Sex	[^68^Ga]Pentixafor				[^18^F]FDG			
			SUV_mean_	SUV_max_	TBR_mean_	TBR_max_	SUV_mean_	SUV_max_	TBR_mean_	TBR_max_
1	54	M	2.3	4.4	1.3	1.0	n/a	n/a	n/a	n/a
2	69	F	2.7	3.9	1.6	1.3	8.0	11.0	4.8	5.4
3	60	M	1.4	2.3	0.9	0.7	8.6	12.0	5.0	5.0
4	78	M	2.5	3.9	1.1	1.0	n/a	n/a	n/a	n/a
5	73	M	2.9	3.6	1.1	0.9	5.8	7.8	3.0	2.5
6	80	M	2.5	3.8	1.6	1.4	11.3	18.4	5.1	5.8>

n/a = not available.

### Immunohistochemistry

**Figure 2:**
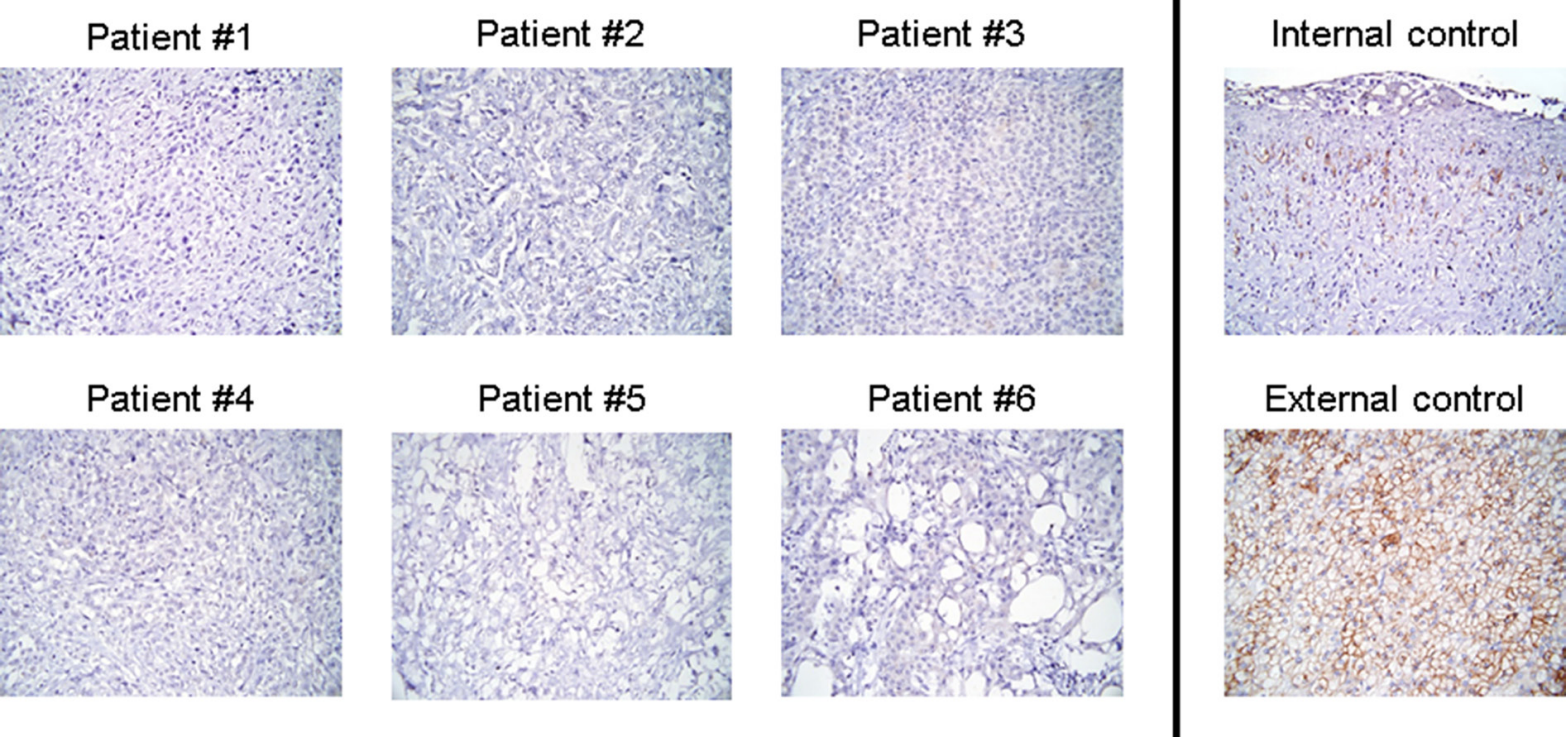
Individual IHC results for CXCR4 Display of the individual results of immunohistochemistry (IHC) for the six patients undergoing [^68^Ga]Pentixafor-PET/CT. In parallel to imaging, no significant CXCR4 expression could be demonstrated on the tumor cell surface. Vascular epithelium served as internal and adrenocortical tissue as external control. Magnification: ×400.

In all patients, imaging results could be compared to CXCR4 expression in biopsies or surgical specimens of the primary tumors assessed by immunohistochemistry. Regarding the histological evaluation of membranous CXCR4 expression, none of samples revealed specific staining for the chemokine receptor (Figure 2).

Nine additional surgical mesothelioma samples from patients not undergoing CXCR4-directed imaging were available for analysis. In parallel with the findings for the patients in whom imaging was performed, all samples were negative for CXCR4 expression in IHC.

## DISCUSSION

This is the first report of *in vivo* imaging of CXCR4 expression in humans with pleural mesothelioma. Whereas a first study on malignant mesothelioma cells (derived from pleural effusions) showed an infrequent chemokine receptor expression [17], a recent report evaluating mesothelioma cell lines and biopsy samples demonstrated a robust CXCR4 receptor expression in malignant PM [5]. Strikingly, IHC for CXCR4 was principally positive in 5 of 6 human mesothelioma cell lines as well as 31/41 mesothelioma tissues. Almost 40% of patient samples demonstrated strong chemokine receptor expression as determined by IHC.

In contrast, in our cohort of PM patients and mesothelioma samples, no significant receptor expression was identified, neither by PET/CT imaging nor immunohistochemical staining. Of note, in the cases with corresponding [^18^F]FDG-PET/CT serving as reference, all tumor lesions exhibited intense FDG uptake (as a marker of tumor viability) with high tumor-to-background ratios. Thus, our findings are in line with a previous study reporting on a rather lower CXCR4 expression profile of solid cancers and metastases (not including mesothelioma) *in vivo* [16]*.* The reason for this obvious discrepancy to the past series is unclear. As a potential explanation, one might argue that cell lines do not fully represent *in vivo* disease, e.g. due to the lack of the tumor microenvironment. The differences in tissue samples might be explained by the fact that receptor surface expression of CXCR4 is a dynamic process which is influenced by a number of factors including therapeutic agents. However, in IHC analyses we did not only find no CXCR4 at the cell surface but also no evidence/indication of intracellular CXCR4 protein expression. In our small cohort, all patients were treatment-naïve at the time of both imaging and biopsy/surgery. Though *Li et al.* report that all patients´ samples in their study were directly obtained from surgery we cannot exclude the possibility of altered CXCR4 expression due to potential previous therapies. Future studies to further investigate therapy-induced down- and –preferably- up-regulation of CXCR4 are highly warranted.

In cell culture experiments, *Li and co-workers* could also demonstrate that CXCL12, the sole ligand of CXCR4, can induce proliferation in mesothelioma which can be antagonized by administration of CXCR4 inhibitors such as AMD3100 [5]. Given the high cellular expression of the chemokine receptor *in vitro* as well as the effects of CXCR4 inhibition, the authors concluded that CXCR4-directed therapies might prove beneficial in high-expressing tumors. Since the advent of [^68^Ga]Pentixafor and its therapeutic counterpart, [^90^Y]/[^177^Lu]Pentixather, theranostic concepts for CXCR4 have successfully introduced by nuclear medicine [18]. Given the high receptor expression previously described, we hypothesized that malignant mesothelioma might represent an attractive target for endoradiotherapy. However, based on our results, CXCR4 does not seem a promising therapeutic option for patients with progressive, irresectable or chemo-refractory disease.

Limitations of the study include that –also due to the low incidence of mesothelioma- only a limited/small number of patients could be included in the study. Therefore, all conclusions have to be drawn with caution, also given the fact that almost all patients and 4/9 samples included in our study represented the epitheloid subtype. Thus, potentially differential CXCR4 expression by different histologic mesothelioma subtypes (e.g. sarcomatoid mesothelioma) might have been missed.

Furthermore, biopsies were not always obtained on a short-term period compared to the time point of PET imaging. However, the maximum interval between imaging and biopsy was 4 weeks with no treatment administered in between. Thus, we are convinced that therapy-induced changes in receptor expression can be ignored.

To conclude, our data suggest a lower frequency of CXCR4-positivity than previously reported. Prior to targeting this chemokine receptor for therapy, robust cell surface expression should be confirmed by immunohistochemistry of the tumor sample, or whole-body [^68^Ga]Pentixafor-PET/CT.

## MATERIALS AND METHODS

### Subjects and research design

Six patients (5 males, 1 female, age 54–80 y; mean, 69 ± 10 y) with histologically proven primary diagnosis of pleural mesothelioma were enrolled.

At the time point of imaging, all patients were treatment-naïve. PET scans were performed for staging purposes ([^18^F]FDG) and to measure the expression of CXCR4 ([^68^Ga]Pentixafor) as a potential therapeutic target for a beta-emitter linked analog. After imaging, surgery was performed in all patients. In five subjects, external beam radiation was performed after the surgical procedure. Two patients also received (platinum-based) chemotherapy. Checkpoint inhibitors were administered in one patient. Detailed patient characteristics are given in Supplementary Table 1.

[^68^Ga]Pentixafor was administered in compliance with The German Medicinal Products Act, AMG §13 2b, and in accordance with the responsible regulatory body (Regierung von Oberfranken). The data analysis was disclosed to the ethics committee of the Universitätsklinikum Würzburg and the need of a formal review was waived. All patients signed written informed consent prior to imaging.

### Surgical mesothelioma samples

In addition to the samples available from the patients undergoing imaging, 9 surgical mesothelioma samples were available for histological analysis.

### Preparation of [^18^F]FDG and the chemokine receptor CXCR4 targeting probe [^68^Ga]Pentixafor

[^18^F]FDG was synthesized in house with a 16 MeV Cyclotron (GE PETtrace 6; GE Healthcare, Milwaukee, USA) using GE FASTlab methodology according to the manufacturer’s instructions.

Synthesis of [^68^Ga]Pentixafor was performed in a fully automated, GMP-compliant procedure using a GRP® module (SCINTOMICS GmbH, Fürstenfeldbruck, Germany) connected to a ^68^Ge/^68^Ga-generator (Eckert und Ziegler, Berlin, Germany) and equipped with a disposable single-use cassette kit (ABX, Radeberg, Germany), using the standardized labelling sequence previously described [19] and 20 µg of unlabelled Pentixafor (SCINTOMICS GmbH). Before use, the radiopharmaceutical was analyzed according to the monographs 2462 (Gallium Chloride) and 2482 (Gallium Edotreotide) of the European Pharmacopoeia. The radiochemical purity was the tracer was > 98% with a specific activity greater than 5 MBq/µg.

### PET imaging

All [^68^Ga]Pentixafor and [^18^F]FDG-PET/CT scans were performed on a dedicated PET/CT scanner (Siemens Biograph mCT 64; Siemens Medical Solutions, Erlangen, Germany) after a 4 hour fasting period. For [^68^Ga]Pentixafor PET, injected activity ranged from 78 to 142 MBq (mean, 123±26 MBq). For standard [^18^F]FDG-PET which was available in 4/6 patients, 287 ± 22 MBq were administered.

Low dose CT scans for attenuation correction were acquired (35 mAs, 120 keV, a 512 × 512 matrix, 5 mm slice thickness with a total of 201 slices, increment of 30 mm/s, rotation time of 0.5 s, and pitch index of 0.8). The imaging field ranged from the base of the skull to the proximal thighs.

Whole-body scans encompassing 6–7 bed positions were performed 1h after administration of the radiopharmaceutical. All PET images were reconstructed using corrections for attenuation, dead-time, random events and scatter. The PET scanner is periodically checked for calibration accuracy as part of quality control according to published guidelines [20].

### Image analysis

Images were visually analyzed by two experienced nuclear medicine specialists (C.L., K.H.). Tumor regions of interest (ROIs) were defined by drawing a standardized 10-mm circular region over the area with the peak tumor activity. Maximum (SUV_max_) and mean standardized uptake values (SUV_mean_) were derived. A reference region was defined by drawing a ROI (diameter of 25 mm) in the cavity of the right ventricle of the heart. The radiotracer concentration in the ROIs was normalized to the injected dose per kilogram body weight of patient to derive the SUVs.

### CXCR4 immunohistochemistry (IHC)

Immunohistochemical analysis of CXCR4 expression was performed on paraffin sections (1 µm) derived from biopsies of the primary tumor using an anti-CXCR4 rabbit polyclonal antibody (ab2074; Abcam, Cambridge, UK) and the DAKO en vision system. For evaluation, the immune reactive score – based on the percentage of CXCR4-positive cells multiplied with the staining intensity- was calculated[12]. CXCR4 positivity of vascular epithelium served as internal and adrenocortical tissue as external positive control, respectively [21–23]. Biopsies were obtained within 4 weeks before/after [^68^Ga]Pentixafor-PET/CT examinations (mean, 20 ± 7 days). In the interval between biopsy/surgery and imaging, no treatment for mesothelioma was administered.

## SUPPLEMENTARY MATERIALS TABLE


