# Supplementary material for: Targeting CXCR4 with [68Ga]Pentixafor: a suitable theranostic approach in pleural mesothelioma?
Source: Oncotarget. 2017 May 27;8(57):96732–7. doi: 10.18632/oncotarget.18235 (PMC5722518; doi:10.18632/oncotarget.18235)
Supplement: Supplementary file 1 [file oncotarget-08-96732-s001.pdf]

Targeting CXCR4 with [^{68}Ga]Pentixafor: a suitable theranostic approach in pleural mesothelioma?

Supplementary Materials

Supplementary Table 1: Patients' demographic data

No.	Age	Sex	Histology	Location	Therapy after PET
1	54	M	Desmoplastic	Left pleura	Surgery, RTx
2	69	F	Epitheloid	Left pleura	Surgery, RTx
3	60	M	Epitheloid	Right pleura	Surgery, RTx
4	78	M	Epitheloid	Left pleura	Surgery
5	73	M	Epitheloid	Left + right pleura	Surgery, RTx, CTx, checkpoint inhibition
6	80	M	Microcystic	Left pleura	Surgery, CTx

CTx = (platinum–based) chemotherapy, F = female, M = male, RTx = radiation therapy.
